# Paving the Way to Unveil the Diversity and Evolution of Phage Genomes

**DOI:** 10.3390/v12090905

**Published:** 2020-08-19

**Authors:** Alejandro Reyes, Martha J. Vives

**Affiliations:** 1 Max Planck Tandem Group in Computational Biology, Department of Biological Sciences, School of Sciences, Universidad de los Andes, Bogotá 111711, Colombia; 2 Department of Pathology and Immunology, The Edison Family Center for Genome Sciences and Systems Biology, Washington University School of Medicine, St Louis, MO 63108, USA; 3 Department of Biological Sciences, School of Sciences, Universidad de los Andes, Bogotá 111711, Colombia

**Keywords:** viral diversity, viral evolution, next generation sequencing, viromes, molecular methods

## Abstract

Phage biology has been developing for the last hundred years, and the potential of phages as tools and treatments has been known since their early discovery. However, the lack of knowledge of the molecular mechanisms coded in phage genomes hindered the development of the field. With current molecular methods, the last decade has been a resurgence of the field. The Special Issue on “Diversity and Evolution of Phage Genomes” is a great example with its 17 manuscripts published. It covers some of the latest methods to sample and characterize environmental and host associated viromes, considering experimental biases and computational developments. Furthermore, the use of molecular tools coupled with traditional methods has allowed to isolate and characterize viruses from different hosts and environments with such diversity that even a new viral class is being proposed. The viruses described cover all different phage families and lifestyles. However, is not only about diversity; the molecular evolution is studied in a set of manuscripts looking at phage-host interactions and their capacity to uncover the frequency and type of mutations behind the bacterial resistance mechanisms and viral pathogenesis, and such methods are opening new ways into identifying potential receptors and characterizing the bacterial host range.

## 1. Introduction

It was not too long ago that we reached a century from the discovery of phages, and since their discovery the phage biology puzzled and enchanted researchers both as a tool and potential treatment due to the highly optimized mechanisms of action that phages require to perform all the functions they have with such a limited coding carrying capacity. Phage biology had its golden age in the 1930s–1950s, where it was fundamental to discover many of the main principles of molecular biology and the development of antibacterial therapies in the eastern European countries. However, given the lack of more sophisticated tools, researchers moved to study antibiotics and other biological systems. In the last decade, with the advancements in sequencing, a whole new view and potential tools to explore phage biology, ecology, and evolution has appeared.

In this Special Issue, we gathered together great examples of the research and science in phage diversity and evolution that has been possible thanks to the advances in Next Generation Sequencing (NGS) and other molecular and computational tools, that are paving the way for both basic and applied research in phage biology. We have divided this editorial into three main sections covering the different studies presented within the Special Issue: methods for studying phages, isolation and characterization of phages, and phage evolution.

## 2. Methods for Studying Phages

The recent resurgence of phage biology studies largely depends on the development and validation of novel methods for phage isolation, characterization, and usage. Phage diversity and ecology studies depend largely on the optimization of such methods. The availability and validation of methods, in particular for an unbiased isolation and purification of viral particles, is of paramount importance. Deng et al. described an optimized protocol for the purification of viral-like particles (VLPs) from fecal samples allowing us not only to characterize the genomic composition of that largely unknown component of the microbiome but to successfully use the isolated phages in further experiments [1]. Such a method will provide a very valuable resource to help increase the reproducibility in the study of the human gut intestinal virome, a field that has been developed in the last decade [2,3,4] and has been the source of many interesting viruses, such as the highly abundant and ubiquitous crAssphage [5].

Characterizing the viral component of the microbiome is complex and doing so without adding significant biases is very important, and while researchers have focused on the viral isolation and sequencing as well as in the bioinformatic analysis [6], an aspect that may have not been deeply studied but could have very important implications is the selection of a mouse vendor and diet in case of mice experiments, such as those demonstrated by Rasmussen et al. [7]. At the end, surprisingly, the vendor had a more significant effect than the diet treatment on both the bacterial and viral components, showing the complexity of any comparison and the importance of describing all the details for the animal experiments.

As is now known, the research of human associated phages is growing rapidly, with many hopes and challenges associated. Many of the these findings and the importance of the study of human-associated phages in the ecological context were very nicely summarized and reviewed by Lawrence et al. where the virus isolation and computational analysis methods are discussed with their corresponding advantages and disadvantages [8]. Complemented with the current knowledge regarding the viromes of the infant and adult healthy gut and the changes caused by Inflammatory Bowel Disease (IBD) and other diseases, we dive further into the potential of virome study in Fecal Microbial Transplants (FMT). Furthermore, even though a significant breath of the human associated phage research has been performed on the gut, an update on the main finding of phages in other sites, such as skin and lungs, was also provided.

If developing and validating experimental methods for the study of the virome is challenging, the refinement of computational methods for studying the virome is a never-ending quest. In our Special Issue we have a very interesting tool called ClassiPhage developed by Chibani et al. where they exploit the increased sensitivity of protein Hidden Markov Models (HMMs) as potential viral markers both for taxonomy and functional annotation [9]. Given the high variability and mutation rates in viral genomes, sequence comparison such as the one performed by Blast limits the capacity to identify related phages only to the close relative ones, at the same species or genus. In the case of ClassiPhage, even though it focused initially on *Vibrio*-associated phages, the method should be easily extendable to other host and viral families. As the phage classification begins incorporating more of the molecular information and distance into the taxonomical hierarchy [10], the development and optimization of HMM-based methods will gain more relevance and importance.

Beyond the method development, the use of such tools for the analysis and composition of novel viruses was an important aspect of this Special Issue, and was well represented by Yang et al. in their effort to characterize the viral composition of the aquatic environment from the South Scotia Ridge, near the Antarctic Peninsula [11]. They were able to show that similarly to other environments, most of the viral diversity was unknown; however, their clustering of environments showed that the Antarctic samples were closer to other open ocean samples than to freshwater samples or other biomes. Additionally, phylogenetic analysis using Terminase or Capsid genes showed that the diversity obtained from those samples included representatives from all major viral lineages.

## 3. Isolation and Characterization of Phages

Moving beyond the characterization of the viromes in different environments, it is now possible to use recently developed tools to isolate novel phages for particular hosts. To start, we had a model organism very thoroughly studied, i.e., *Escherichia coli*, where it is still possible to isolate and identify novel phages such as the 50 new phages identified by Korf et al. from different sources [12]. The newly isolated viruses showed variation in their host range associated to the viral family, Myoviridae having wider host range than Siphoviridae phages. Furthermore, using VICTOR [13] for phylogenetic inference it was possible to propose seven new genera and subfamilies based on current and discovered viruses; of particular interest was the PTXU04 virus which does not have any close relative in a public databases, or the jumbo Goslar phage with a genome size of ~230 Kb.

However, not only *E. coli* benefits from phage discovery. Sampling in Thai poultry farms, Phothaworn et al. were able to isolate 108 phages that infect several strains of *Salmonella enterica* serovar Typhimurium; most of these phages were flagellotropic chi-like viruses, a temperate viral genus that has been isolated in western countries [14]. Those results show the global distribution and ease of dissemination of viruses and their hosts. In parallel, as a Thai poultry were shown to be the perfect scenario for the discovery of *Salmonella* phages, a lake in China was shown to be ideal for the recovery of nine new phages capable of infecting *Aeromonas*, as shown by Bai et al. where a very thorough phenotypic and genotypic characterization of the previously unknown viruses was performed [15]. Results suggests, as observed in other hosts, that the genetic diversity of phages capable of infecting *Aeromonas* is extremely high with clusters of phages sharing no genes or sequence similarity between them but still able to infect the same bacterial host.

This viral diversity is not only common for model organisms and well-studied hosts. The large genetic diversity of viruses and the plethora of uncharacterized and hypothetical genes commonly referred as the “viral dark matter” is common in all bacterial hosts. An example of this was shown by Xi et al. and their characterization of vB_AviM_AVP, a novel phage and the first one reported to infect *Aerococcus viridans* [16]. This virus showed no close similarity to any other reported viruses, and their preliminary analysis suggests that it could potentially constitute a new viral class. Defining new viruses is not only about sequencing but also the functional characterization and mechanism of interaction with the corresponding bacterial host. Attai et al. were able to isolate from residual waters and characterize a temperate and a lytic phage capable of infecting *Agrobacterium tumefaciens* [17], a very important agricultural and biotechnological bacterium. The functional characterization included the use of the portal vertex protein of the lytic virus to classify it as part of the T4-like phages, while the terminase on the temperate phage allowed the classification as a T7-like phage.

Finally, returning to the well-characterized hosts with still a lot of the viral space to explore, as in *E. coli*, *Bacillus* is a genus with many described phages but with enormous potential for more discovery. In our Special Issue, Yuan et al. showed that the novel virus vB_BthS_BMBphi infecting *Bacillus thuringiensis* is distantly related to other known *Bacillus* phages [18]. A particular effort was made in characterizing the phage endolysin where regardless of the very narrow host range that the phage exhibits, the endolysin was capable of lysing a wide range of members of the *Bacillus cereus* group, with potential biotechnological applications. This was not the only analysis done on a *Bacillus* phage: showing the interest in the bacilli group and its potential for phage diversity exploration, Fu et al. analyzed 389 candidate prophages with 135 potential complete prophages, finding higher diversity than those found in other bacteria such as *Pneumococcus* and *Desulfovibrio* [19]. Furthermore, the viability of the prophage was tested for induction with Mitomycin C finding that 7/10 phages were successfully induced. This diversity of prophages was also confirmed at the bacterial strain level, with the capability of phages for harboring different antibiotic resistance genes. Additionally, as we learn more of the phage lifecycles, we realize that lysis or integration within the host genome are not the only alternatives. The phages in *Bacillus* were not only capable of inserting into the bacterial genome, they were also capable of remaining in the cytoplasm as plasmids. This was shown by Piligrimova et al. who show that the vB_Bts_B83 phage, which was previously thought to be a plasmid, actually represents a new member of the Siphoviridae family, confirmed by electron microscopy and different molecular validation tools [20].

## 4. Phage Evolution

A Special Issue on diversity an evolution had to contain great examples of viral evolution and adaptation. This was not the exception, where four different studies showed the potential of how new technologies and NGS can be effectively used to study phage evolution and phage–host interaction. Our first example uses a model organism such as *Bacillus thuringensis*. Yuan et al. performed a variant analysis on the bacteria and its corresponding phages and demonstrated that the mutations obtained were directly involved in phage–host interaction [21]. On the bacterial side, the mutations were diverse and occurred in different genomic locations but tended to be conserved in different bacterial populations and were associated with cell wall synthesis, flagellum synthesis, and other known reported phages receptors. On the phage side, mutations were few and highly conserved, occurring in three different tail-associated genes. This limited and specific number of mutations suggest a co-evolution dynamic denominated “dying to survive”, where the phage needs to make many potentially deleterious mutations, likely leading to loss of infectivity in order to accumulate at least one mutation that will potentially overcome bacterial resistance.

A similar finding regarding the mutations of the tail genes was observed in a forced co-evolution of two phages from the genera Felixunavirus when challenged for 12 h with their host, *Salmonella* Infantis. Rivera et al. were able to identify the molecular changes along with phenotypical changes on both phages and the bacterial host unveiling the molecular mechanisms of resistance in the bacteria and its corresponding evasion by the phage [22]. The phage with a broader host range accumulated a larger number of mutations, while the narrow range phage was capable of doing a host switch after the 12 h of interaction.

In a similar system using *Salmonella* Enteritidis, Holguin et al. evaluated the co-evolution of the *Salmonella* phages under different scenarios, using either a single phage-host, multiple phages in one host, or phages and antibiotics [23]. This setup showed that there was no antagonistic co-evolution among the phage and the host, and the phages isolated from each microcosm were infective only against the ancestral strain. Similar to the other co-evolution experiments, on the bacterial side the mutations were identified mainly in the cell wall or capsule genes, and surprisingly, no mutations were accumulated on the CRISPR loci. Importantly, the analysis performed allowed the suggestion of the protein BtuB as the potential phage receptor for the phage under study, another important outcome of phage evolution studies. On the phage side, no mutations were identified that could be associated with a capacity of infecting resistant bacteria and in general, mutations reduced its infectivity.

Our final example of phage evolution was performed studying marine *Synechococcus* phages. Kupczok and Dagan measured the rates of molecular evolution by evaluating mutations and recombination events in phage genomes, where the latter seem to have a more significant effect in short-term variation [24]. They concluded that the RIM8 linage follows a molecular clock model and found that the substitution rate is 10-fold less than the one reported for other cyanophages in different environments. In this study, the signal of recombination is recognized by the co-localization of variable genes (present in less than 10 genomes) and likely associated to genes being mobilized from the bacterial host, and thus, capable of conferring metabolic benefits to the current or potentially new bacterial hosts.

## 5. Perspectives

Phage diversity and evolution are exciting fields, where each new report is interesting and represents an amazing avenue for areas to explore. Developments in techniques and analysis tools are of paramount importance to allow these new data to emerge. We are experiencing the revival of the interest and perspectives of applications of phages, and we are bound to build it on solid grounds. The future is open for phage diversity and evolution studies, with questions as old and wide as how big is phage diversity, what are the impacts of phages in the ecosystems, how can we take advantage of this diversity, and how safe is it? We, as biologist, microbiologists, data scientists, and biotechnology-driven scientists, are eager to witness the answers to these questions.
